# Designing a rabies control mobile application for a community-based rabies surveillance system during the COVID-19 pandemic in Bali, Indonesia

**DOI:** 10.14202/vetworld.2022.1237-1245

**Published:** 2022-05-21

**Authors:** I Made Subrata, Ngakan Putu Anom Harjana, Kadek Karang Agustina, Sang Gede Purnama, Made Pasek Kardiwinata

**Affiliations:** 1 Department of Public Health and Preventive Medicine, Faculty of Medicine, Udayana University, Denpasar 80225, Bali, Indonesia; 2 Center for Public Health Innovation, Faculty of Medicine, Udayana University, Denpasar 80225, Bali, Indonesia; 3 Department of Veterinary Public Health, Faculty of Veterinary Medicine, Udayana University, Denpasar 80225, Bali, Indonesia

**Keywords:** community-based intervention, One Health, post-study system usability questionnaire, rabies, surveillance system

## Abstract

**Background and Aim:**

Rabies remains a public health concern in Indonesia, and the coronavirus disease (COVID-19) pandemic has stymied rabies prevention and control efforts. There is a need to transform the rabies program to be adaptable to pandemic situations to improve program coverage on dog vaccination and rabies surveillance. This study aimed to create a rabies control (RaCon) mobile application for a community-based rabies surveillance system during COVID-19 in Bali, Indonesia.

**Materials and Methods:**

We employ the Design Science Research methodology. Surveillance officers, veterinarians, community leaders, outreach workers, and dog owners participated in a series of offline in-depth interviews and focus group discussions. The RaCon prototype was evaluated using the Post-Study System Usability Questionnaire (PSSUQ) framework, which included the system’s usefulness, information quality, and interface quality. In this study, we used both a qualitative (n=50) and quantitative (n=342) approach.

**Results:**

According to the findings of this study, integrating public health and animal health into the rabies surveillance system are critical to supporting the One Health approach and encouraging community engagement in rabies programs. The RaCon prototype is expected to include features such as pet ownership, case report, news and announcements, nearest vet, health information, outbreak radar, emergency call, and app feedback. The RaCon prototype passed both qualitative and quantitative evaluations, indicating that it could be used to support the rabies surveillance system, particularly in the COVID-19 situation.

**Conclusion:**

The RaCon prototype was accepted by the users and got positive feedback in terms of the system’s usefulness, information quality, and interface quality dimension. As a result, this prototype has the potential to be integrated into the rabies surveillance system in Bali, particularly to strengthen the community-based rabies surveillance system. Even though this prototype received positive feedback, this study focuses solely on the design development and evaluation of its user interface. As a result, further development is required before incorporating RaCon into the rabies prevention and control program.

## Introduction

Rabies remains a public health concern in Indonesia, with domestic dogs accounting for the vast majority of rabies cases [1]. In 2020, 32 out of 34 provinces reported bite cases from rabies-infected animals (e.g., dogs, cats, bats, and monkeys), with 40 rabies-confirmed deaths [2]. In comparison to other parts of Indonesia, Bali Province has had the most bite cases since 2018, with over 26,000 bites reported each year. Rabies cases were discovered in all nine districts of Bali Province until the end of 2020 [3].

A high number of bite cases in Bali were linked to increased dog ownership [4]. Historically, the Balinese people allowed their dogs to roam freely around the streets [5]. This situation raises the risk of rabies transmission among dogs, while also posing a threat to humans. The first case of rabies in humans and dogs was discovered in Bali in 2008, and over 130 people died from the disease between 2009 and 2011 [6]. As rabies spread across the island, the government declared an outbreak of rabies.

The government has provided a post-exposure rabies vaccine for humans, a dog vaccination program, and mass culling for unconfined dogs to control rabies transmission [6]. Because of the low cost of the sterilization program, dog culling remains an option, whereas the sterilization program is only available to owned dogs (pets). Although increasing dog vaccination coverage is critical for rabies control (RaCon), implementation can be difficult, and population turnover erodes coverage [7]. For the past decade, there has been a campaign to confine the dogs and provide a free dog vaccination program [1,7]. Furthermore, community involvement in rabies programs, such as surveillance and health promotion activities aimed at increasing rabies knowledge and awareness, has begun in some villages [8]. These efforts have resulted in high vaccination coverage (83.6%) and more dogs being confined [7].

While the government in Bali struggled to meet the target of eliminating rabies in all nine districts by the end of 2020, the coronavirus disease (COVID-19) pandemic slowed the rabies prevention and control program’s progress. Before the COVID-19 pandemic, mass dog vaccinations were easily carried out during village community gatherings. Furthermore, community members’ door-to-door dog mapping is becoming a routine activity to strengthen rabies surveillance [8]. During COVID-19, the dog vaccination program and rabies surveillance were hampered due to social distancing and a lockdown policy in Bali for preventive measures. A similar situation was also discovered in the rabies prevention program during the COVID-19 situation in Arequipa, Peru [9].

As a result of the social restrictions imposed during the COVID-19 situation, it was necessary to transform the rabies prevention and control program in Bali to meet the needs of COVID-19 situations. In response to the COVID-19 situation, digital-based technology such as telemedicine and other mobile applications has emerged to date [10]. These digital-based technologies have empirically strengthened community outreach, improved data recording, and reporting quality, and served as a tool for detecting zoonotic diseases [11-13].

There are currently no published studies in Indonesia that explore the use of digital-based technology for rabies prevention. This study aimed to create a mobile application for RaCon in Bali, Indonesia.

## Materials and Methods

### Ethical approval and Informed consent

The study was approved by the Research Ethics Commission, Faculty of Medicine, Udayana University/Sanglah General Public Hospital, Bali Province, Indonesia (No. 1996/UN14.2.2.VII.14/LT/2021). Participants in this study were voluntary and anonymous. For informants involved in in-depth interviews and focus group discussions, written informed consent was obtained. For the online survey, digital informed consent was obtained before participants completed the online form. In addition, participants received IDR 150,000 for involvement in the qualitative study and IDR 25,000 phone credit if they completed the questionnaires in the online survey.

### Study period and location

This study was conducted from June to November 2021. The offline data collection for in-depth interviews and focus group discussions was conducted in Denpasar, Badung, Gianyar, and Klungkung. While the online data collection for the quantitative survey was conducted in all nine districts of Bali Province.

### Study design

The Design Science Research approach was used in this study, which includes problem identification and objective definition, design and development, demonstration, evaluation and communication, and conclusion [14]. We conducted offline in-depth interviews and focus group discussions with relevant stakeholders during the problem identification and objective definition stages. The data gathered during the problem identification process were then used to design and develop the prototype. We tested the prototype with relevant stakeholders and population targets during the demonstration stage. The prototype was then evaluated using qualitative and quantitative methods, and it was refined to produce the final model.

### Data collection procedure

The prototype development and evaluation process was conducted in two iterations. The activity diagram was used in the first iteration, which allows users to test the information flow in the system. We conducted offline in-depth interviews with veterinarians, surveillance officers from the district and province levels, and community leader representatives (n=10 participants). We also held four focus group discussions involving dog owners and outreach workers (n=40 participants). The outreach workers were hired through Program Dharma, a community-based initiative for the rabies project in Bali [8]. These informants were selected using purposive sampling and they were involved in the entire prototype development. The information gathered during in-depth interviews and focus group discussions included the existing rabies surveillance system in Bali, the model of community-based intervention in the rabies program, and need assessments for prototype development.

We create a prototype that can be accessed through the Android and iOS operating systems in the second iteration. The Post-Study System Usability Questionnaire (PSSUQ) was used in the second iteration of the evaluation process to evaluate three dimensions: System usefulness, information quality, and interface quality [14]. PSSUQ was made up of 19 items that have a 7-point Likert scale. If the average of all dimensions was greater than 4-point (median), the prototype is considered good. The PSSUQ evaluation was carried out through an online survey involving 342 participants who were also selected using purposive sampling.

### Statistical analysis

Thematic analysis was used to analyze the qualitative data in this study, while STATA 15 (StataCorp LLC, USA) was used to analyze quantitative data for respondents’ demographic characteristics and PSSUQ evaluation.

## Results

### Problem identification and objective definition

In-depth interviews and focus group discussions were held with a total of 50 participants to understand better the existing rabies surveillance system in Bali, the model of community-based intervention in the rabies program, and need assessments for prototype development.

#### Existing rabies surveillance system in Bali

This study discovered that rabies surveillance activities and programs for humans (or public) and animal health were separated at the district and provincial levels. As a result, data collection and reporting for rabies cases were not integrated between public and animal health. The rabies surveillance system for public health uses an early warning and response system from the Ministry of Health, Republic of Indonesia (https://skdr.surveilans.org/), while the animal health surveillance system uses iSIKHNAS (https://www.isikhnas.com/) from the Ministry of Agriculture, Republic of Indonesia. Because the data are only accessible to authorized surveillance officers, data sharing for decision-making and program planning for rabies prevention and control were difficult to be conducted.

“*Only surveillance officers have access to the system’s data. So, far, anyone or any organization that requires rabies data must make an official request to us*” *–* public health surveillance officer at the province level (male)

“*There was no open-access dashboard to show the distribution of rabies cases. As a result, other Dinas (government agencies) must request the data via an official letter*” *–* animal health surveillance officer at the province level (male)

This study also discovered that the existing rabies surveillance system primarily supports passive surveillance activities, with active case detection relying on reports from surveillance officers in *Puskesmas* (public health center) and *Puskeswa*n (animal health center). The current animal health surveillance system still does not include data from dog vaccinations and veterinary examinations performed by an independent practice veterinarian. Furthermore, the current animal health surveillance system does not accommodate the role of dog owners in providing data on the number of dogs owned and their vaccination status. Underreporting occurs due to this situation, affecting the validity of vaccination coverage.

“*I never report the rabies vaccinations I perform in my private clinic. I only keep the data for my personal use*” *–* Veterinarian in Denpasar (male)

“*Based on what I knew, they (the vaccinator officer) only report the dog vaccinations performed in bale banjar (mass dog vaccination program). Furthermore, there is no requirement to report it*” *–* Veterinarian in Klungkung (male).

#### Community-based intervention in the rabies program

We conducted the focus group discussion because some villages in Bali have initiated a community-based intervention for rabies prevention and control program called Program Dharma, and we wanted to understand the workflows, strengths, weaknesses, opportunities, and threats. We discovered that this community-based intervention empowers village residents to be involved in the rabies program through activities such as door-to-door mapping for owned and stray dogs, community education about rabies, facilitating dog vaccination programs, and serosurveillance of rabies, and advocating for the decision-making process for regulations and program planning. Furthermore, the outreach worker stated that a community-based intervention in the rabies program could strengthen the surveillance system of rabies at the village level because the data from dog mapping could provide a more accurate number of dog population and vaccination coverage at village levels. These are the advantages of a community-based intervention strategy for rabies prevention and control.

“*The program’s implementation (a community-based rabies intervention) is excellent. We can get a more accurate count of the number of stray dogs in our village. We can also find out how many dogs have been vaccinated, who owns them, and how healthy they are*” *–* Outreach worker in Sanur Kauh (male)

“*Using a cellphone application to track the movements of dog owners and stray dogs. It’s called KoboToolbox. We can map the GPS coordinates and photograph the dogs. As a result, we have a map of the distribution of dogs as well as their vaccination status*” *–* Outreach worker in Sibang Kaja (male)

KoboToolbox is an open-source data collection tool. Related to rabies surveillance, the tools used in Program Dharma already provided a solid model of a community-based rabies surveillance system that enables participatory disease detection and prevention. Those tools, however, are standalone and only applicable at the village level. As a result, increasing the use of this tool at district or provincial levels becomes a good way to support the rabies surveillance system. Integrating a community-based surveillance approach with the existing surveillance system could be an effective strategy for improving rabies prevention and control programs.

“*I believe it would be fantastic if the Program Dharma model could be adopted in other villages in Bali to combat rabies. The monitoring tools, in particular, made use of smartphone-based applications. It simply needs to be redeveloped so that it can be integrated with existing programs in other related Dinas (government organizations)*” *–* Community leader representatives in Sibang Kaja (female)

“*I believe that related Dinas should consider using the system used by the Program Dharma in dog mapping. If Dinas can design a system that is at least similar to the one currently in use here and can be integrated, I believe that the rabies program report will be even better*” *–* Community leader representatives in Sanur Kauh (male)

#### Need assessments for prototype development

We identify the feature requirements for the RaCon mobile application prototype based on the existing rabies surveillance system and the community-based rabies intervention approach. According to the surveillance officer, it is critical to develop a system that can provide output for cross-sectoral collaboration. The system was expected to provide accurate data on bite cases, dog population, vaccination coverage, and distribution of rabies cases.

“*If there is a system that can integrate animal and human health data, I think it will facilitate cross-sectoral coordination in rabies prevention and control efforts. We will no longer need to bother with correspondence and the bureaucratic process may be simplified, allowing for more effective communication between related Dinas because we already use single data*” *–* Public health surveillance officer at the province level (male)

Like the surveillance officer, the veterinarian expected that the system that would be developed would make the process of data recording and reporting related to dog vaccination and disease detection among dogs or other animals easier.

“*It would be preferable if the system developed, whether in the form of a mobile application or a website, could assist us in the process of data recording for dog vaccination in our private clinic as well as to make it easier for us to provide the report to the Dinas*” *–* Veterinarian in Badung (male)

The outreach workers anticipated that the system that will be developed would also serve as a communication platform for rabies prevention and control, including in an emergency such as a dog bite. Outreach workers also anticipated that the features already available in the KoboToolbox used in Program Dharma would be enhanced to support the rabies surveillance system.

“*Based on our previous dog mapping experiences, features such as GPS tracking and picture tagging proved useful in monitoring dog distribution. Another challenge is that when someone is bitten by a dog, it is difficult to locate Puskesmas’ emergency contact information. As a result, it will be better if the system that is being developed provides a list of emergency contacts from Puskesmas and other related stakeholders*, *so we can deal with dog bite cases quickly to avoid fatalities*” *–* Outreach worker in Sanur Kauh (male)

On the other hand, the dog owners expected that the system that would be developed would be accessible to them so that they could obtain information about rabies and other zoonotic diseases. Furthermore, they want to participate in the rabies prevention and control program by providing information about the number of dogs they own and their vaccination status. In terms of access to veterinary services, dog owners also expected that the system could provide information about the nearest vet clinic or pet shops. These are the features that were expected to be available in the prototype.

“*I want to protect my dog from rabies. As a result, knowing about rabies is essential for me and my neighborhood. I will be thrilled if I could get that kind of information on my phone*” *–* Dogs owner 1 (male)

“*I always forget about the rabies vaccination history of my dog. Can that mobile app provide a reminder when it’s time to get the vaccination shoot for my dog?*” *–* Dogs owner 5 (male)

“*Sometimes, I feel difficult to take care of my sick dog because the vet that I usually visit is closed. I also don’t know where I can get veterinary care. As a result, it would be preferable if the application developed could provide a list of nearby veterinary services as well as their availability*” *–* Dogs owner 4 (female)

### Design and development

#### First iteration

**Figure-1:**
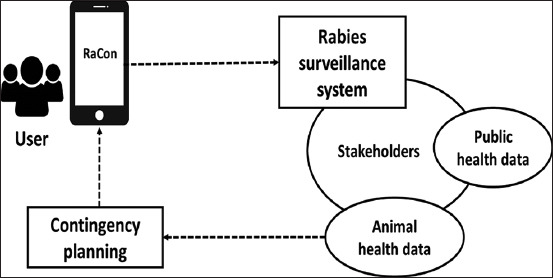
Activity diagram for the modified rabies surveillance system.

For the modified rabies surveillance system, we created an activity diagram (Figure-1). This activity diagram is refined and validated through in-depth interviews and focus group discussions with the same participants who have been involved since the problem identification process began. This activity diagram depicts the information flow from villages to province levels between users (e.g., dog owners, veterinarians, and community members) and stakeholders, particularly in developing a contingency plan for improving rabies prevention and control programs. Compared to the existing rabies surveillance system implemented in Bali, this modified activity diagram integrates the public health data and animal health data. The data generated by this system could be used in the decision-making process related to rabies prevention and control programs. This will give benefits to all, especially the users.

#### Second iteration

**Figure-2:**
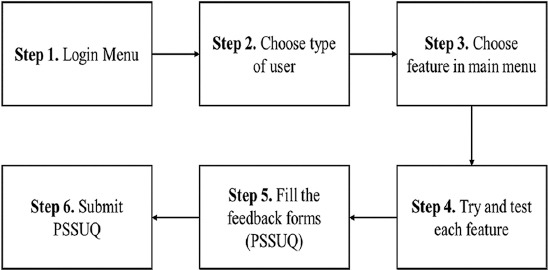
Workflow of rabies control (RaCon) prototype. RaCon=Rabies control.

**Figure-3:**
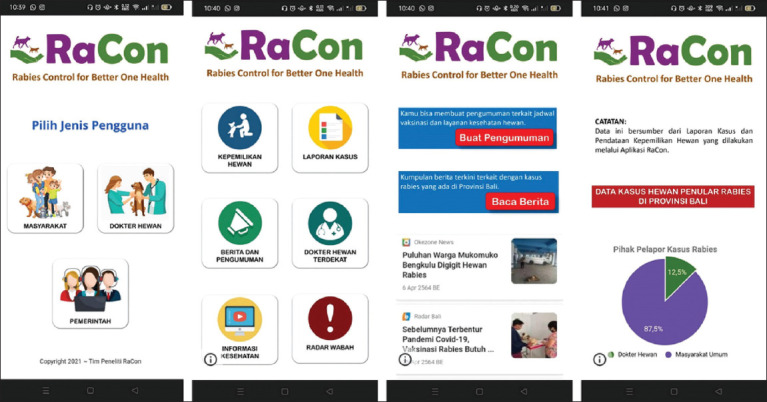
Example of the user interface of RaCon prototype (Steps 2, 3, and 4). RaCon=Rabies control.

In this second iteration, we create a prototype for RaCon. The prototype’s workflow consisted of six steps (Figure-2). The first step is for the user to access the login menu by entering a username and password. In the second step, the user must select the type of user, which includes the public, veterinarian, and surveillance officer. The users must then select from the available features. Because of various roles in the rabies surveillance system, the features will differ depending on the type of user. The RaCon prototype included eight features: Pet ownership, case report, news and announcements, nearest vet, health information, outbreak radar, emergency call, and app feedback. All of these features can be accessed through https://bit.ly/AppRaCon. We created the prototype using Google Sites (https://sites.google.com/) combined with the database using KoboToolbox (https://www.kobotoolbox.org/). Both of these platforms were open sources, which meant that they could be accessed from any smartphone operating system without the need to install the application. An example of the user interface of the RaCon prototype is depicted in Figure-3.

### Demonstration, evaluation, and communication

Following the development of the RaCon prototype in the second iteration, we tested the prototype through an online survey of community members, both dog owners, and non-dog owners. We shared the prototype (https://bit.ly/AppRaCon) on social media, such as Facebook, Instagram, and WhatsApp. We invited the general public to test the RaCon prototype and provide feedback. Those who were 18 years old or above and had access to the internet through Android or iOS smartphones were considered eligible. The RaCon prototype was demonstrated and evaluated following the procedure depicted in Figure-2. Participants who completed the PSSUQ were compensated with IDR 25,000 in phone credit.

**Figure-4:**
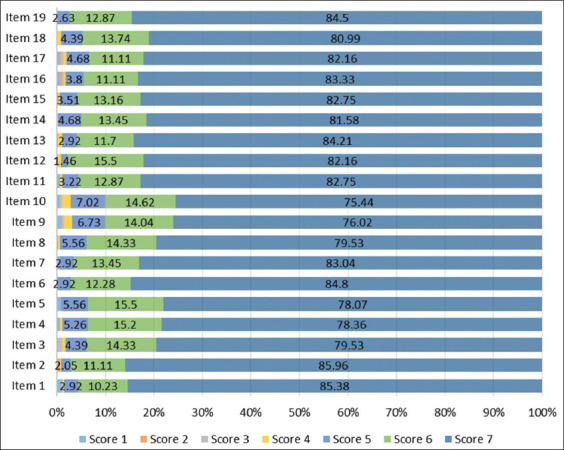
Distribution of participants’ responses on PSSUQ item (n=342). PSSUQ=Post-Study System Usability Questionnaire.

There were a total of 342 participants who submitted the complete PSSUQ from the online survey (Table-1). More than half of the participants were female and aged ≤25 years old. The majority of participants resided in the districts of Denpasar and Gianyar. Furthermore, more than 80% of the participants were dog owners or public users. Furthermore, health information becomes the most popular feature in the RaCon prototype. PSSUQ was used to evaluate the prototype based on system usefulness (items 1-8), information quality (items 9-15), and interface quality (items 16-19). The PSSUQ for this study can be found in Supplementary Table-1 and was adopted from a previous study [14]. The average score for all dimensions in this study was 6.74, indicating that participants were generally satisfied with the RaCon prototype (Table-2). Figure-4 depicts the participants’ responses to each PSSUQ item.

**Table 1:** Demographic characteristics of participants and favorite feature of RaCon prototype (n=342).

Characteristics	Frequency	Percentage
Gender		
Male	129	37.72
Female	213	62.28
Age group		
≤25 years old	265	77.49
>25 years old	77	22.51
Place of resident		
Badung	43	12.57
Bangli	8	2.34
Buleleng	47	13.74
Denpasar	86	25.15
Gianyar	85	24.85
Jembrana	14	4.09
Karangasem	19	5.56
Tabanan	24	7.02
Outside Bali	16	4.68
Dog ownership		
No	34	9.94
Yes	308	90.06
Type of user		
Public	301	88.01
Veterinary	23	6.73
Surveillance officer	18	5.26
Favorite feature of RaCon app		
Pet ownership	72	21.05
Case report	15	4.39
News and announcements	39	11.40
Nearest vet	46	13.45
Health information	143	41.81
Outbreak radar	21	6.14
Emergency call	6	1.75

RaCon=Rabies control

**Table 2:** PSSUQ values (n=342).

System usefulness	Information quality	Interface quality	Average score	Note
6.74	6.72	6.75	6.74	Above the median (4) is considered good

PSSUQ=Post-Study System Usability Questionnaire

Furthermore, the communication process was performed to evaluate the prototype using a qualitative approach, with similar participants from problem identification stages involved. We discovered that participants thought the RaCon prototype could be useful for rabies prevention and control programs, particularly during the COVID-19 pandemic. The RaCon prototype’s available features could facilitate teleconsultations and increase veterinary services, even in pandemic situations where social distancing policies were used. Furthermore, the RaCon prototype allows dog owners to participate in an active rabies surveillance system, indicating good progress in strengthening rabies prevention and control programs.

“*The concept of the remote consultation in RaCon is good. It solves the problem of some veterinary services being closed, so the community can still access the services from their homes even during COVID-19*” *–* Veterinarian in Badung (male)

“*I imagine that this app will help dog owners like me to get a rabies vaccination for my dog, especially since the pandemic is over. I mean, we don’t have to wait for the mass dog vaccinations that are usually held in bale banjar. I recommend that the vaccinator use this app to perform door-to-door dog vaccinations*” – Dogs owner 4 (male)

“*Because the COVID-19 situation limits us outreach activity, I am concerned that the rabies program that we have worked so hard to develop will be rendered ineffective if vaccination coverage is reduced. If the dog owners can record the vaccination status of their dogs in RaCon, it will help us to monitor the vaccination coverage*” *–* Outreach worker in Sibang Kaja (male)

## Discussion

This study aimed to create a mobile application for RaCon in Bali, Indonesia, to meet user expectations for usability, particularly during the COVID-19 situation. The application was created to assist stakeholders involved in the rabies prevention and control program in reaching their goals. Furthermore, the application is intended to strengthen the rabies surveillance system, particularly by integrating public health and animal health data and encouraging dog owners to participate in disease detection. A study in Haiti discovered that the community-delivered SMS alerts could increase the awareness of dog owners about rabies and increase their participation in mass dog vaccinations [15]. Another study in India discovered that the use of GPS and smartphone technology for rabies mapping has enabled efficient team management as well as real-time data entry and analysis [16]. These findings suggested that digital technology, such as the RaCon application could help community-based rabies prevention and control programs.

Because the design of the surveillance system in this study promotes the integration of public health and animal health surveillance systems, this effort may aid in the implementation of the One Health approach to rabies prevention. For the past decade, Bali’s rabies prevention program has followed the One Health approach, particularly by encouraging cross-collaboration between stakeholders in the medical and veterinary sectors [17]. The One Health approach was considered the most effective method of RaCon [18]. A strong surveillance system is required to prevent zoonotic diseases that could become outbreaks, such as rabies. In this regard, a study in Thailand discovered that using digital technology in participatory disease detection using the One Health approach could increase community empowerment in disease detection and prevention [19]. According to these findings, the RaCon application has the potential to improve the rabies surveillance system in Bali, particularly by promoting the One Health approach and community engagement.

The features in the RaCon application were designed to transform traditional rabies programs to adaptable in the COVID-19 situation where online-based activity became a priority to facilitate the One Health approach for rabies prevention and control. To monitor the dog populations, vaccination status, and diseases detection, the RaCon application includes pet ownership and case report feature. It was anticipated that by encouraging community members, particularly dog owners, to participate in active rabies surveillance, the surveillance data quality would improve. This approach was similar to previous research on the use of digital technology in community-based rabies prevention and control programs [11,12,15,16,19].

A public campaign to increase rabies knowledge and awareness was also necessary [20]. To aid this effort, the RaCon application has been enhanced with features such as news and announcements, health information, and outbreak radar. The information in this feature may have been provided by related stakeholders such as the public health and animal health departments, researchers, experts, as well as professionals. A public campaign utilizing digital technology such as the RaCon application could broaden the targeted audience, thereby improving rabies knowledge and awareness [21]. Furthermore, the use of a GPS tracking system in the RaCon application could create an outbreak radar, which contains real-time updates on rabies cases in specific areas. This outbreak radar will notify users when users travel to a high-risk area (e.g., high number of stray dog populations, high rabies cases, and low dogs vaccination coverage). As a result, users should be prepared to manage the rabies risk when visiting certain areas [22].

It is challenging to provide veterinary access, such as dog vaccination and rabies detection, during COVID-19 [9]. In response to this situation, the RaCon application is also designed to provide remote veterinary services through the nearest vet and emergency call features. Because the demand for veterinary services was high during the pandemic, it was critical to provide both access and affordable veterinary services [23]. RaCon application allows users to locate the nearest vet clinics based on their requirements. Furthermore, the emergency call feature could be used in the case of an emergency, such as bites or scratches from rabid animals. These features were expected to reduce rabies-related fatalities in humans and animals. The PSSUQ evaluation of the RaCon prototype found that participants were satisfied with the system on the dimensions of usefulness, information quality, and interface quality. While the system’s usefulness assesses whether the system can provide benefits to users concerning a specific task, information quality assesses whether the system can be easily understood and effectively assist in the completion of tasks [14]. On the other hand, the interface quality is related to whether the system is capable of satisfying its users [14]. This indicated that the final RaCon prototype, obtained after two iterations, was well received by target users. The qualitative evaluation confirmed the PSSUQ results, indicating that the RaCon prototype was feasible to implement to support the rabies surveillance system, particularly during the COVID-19 situation.

There are several limitations to this study. Even though the RaCon prototype received positive feedback, improvements could be made before implementation. This research only looks at the design and evaluation of the user interface. The study to determine whether the data collected in this application met the needs of the surveillance report is critical to ensuring that the RaCon application meets the users’ needs. Furthermore, before implementing RaCon, a feasibility study is required to understand the product feasibility, market feasibility, organizational feasibility, and financial feasibility of the implementing units. This will be essential to determine improvements for the system to improve its usability [24]. Another limitation was related to participants’ distribution which was dominated by young age (≤25 years old). As a result, this study is unable to depict the user’s perception from an older age (>25 years old). The online survey method used in this study could have an impact on this. As a result, future research should target the older age population to comprehensively understand the RaCon prototype acceptance.

## Conclusion

As the COVID-19 pandemics slowed the progress of the rabies prevention and control program in Bali, efforts to transform the rabies program are critical. It was critical to integrate the public health and animal health surveillance systems based on the identification of the existing rabies surveillance system that had been implemented in Bali. This will help to support the One Health approach while also encouraging community participation in rabies prevention and control programs. Furthermore, this research yields a RaCon prototype that can be accessed by the general public, veterinary professionals, and surveillance officers. Pet ownership, case report, news and announcements, nearest vet, health information, outbreak radar, emergency call, and app feedback are among the eight features of the RaCon prototype. The demonstration and a quantitative and qualitative evaluation of the prototype show that the participants were satisfied with the RaCon prototype, particularly in terms of system usefulness, information quality, and interface quality. As a result, before the RaCon mobile application is implemented in rabies programs, a feasibility study is required to understand the areas that require improvement.

## Authors’ Contributions

IMS and NPAH: Conceived the idea. NPAH: Analyzed the data. NPAH: Drafted the manuscript. MS, NPAH, KKA, SGP, and MPK: Critically reviewed and edited the manuscript. All authors have read and approved the final manuscript.

## Supplementary

**Supplementary Table-1:** Post-Study System Usability Questionnaire (PSSUQ) items

S. No.	PSSUQ item	1	2	3	4	5	6	7
1.	Overall, I am satisfied with how easy it is to use this system.							
2.	It was simple to use this system.							
3.	I could effectively complete the tasks and scenarios using this system.							
4.	I was able to complete the tasks and scenarios quickly using this system.							
5.	I was able to efficiently complete the tasks and scenarios using this system.							
6.	I felt comfortable using this system.							
7.	It was easy to learn to use this system.							
8.	I believe I could become productive quickly using this system.							
9.	The system gave error messages that clearly told me how to fix problems.							
10.	Whenever I made a mistake using the system, I could recover easily and quickly.							
11.	The information (such as online help, on-screen messages, and other documentation) provided with this system was clear.							
12.	It was easy to find the information I needed.							
13.	The information provided for the system was easy to understand.							
14.	The information was effective in helping me complete the tasks and scenarios.							
15.	The organization of information on the system screens was clear.							
16.	The interface of this system was pleasant.							
17.	I liked using the interface of this system.							
18.	This system has all the functions and capabilities I expect it to have.							
19.	Overall, I am satisfied with this system.							

Score 1=Strongly disagree, Score 7=Strongly agree, system usefulness (items 1-8), information quality (items 9-15), interface quality (items 16-19). PSSUQ=Post-Study System Usability Questionnaire
